# Association between Muscle Mass Index and Neurological Outcomes of Patients with Out-of-Hospital Cardiac Arrest

**DOI:** 10.3390/life14060680

**Published:** 2024-05-24

**Authors:** Yongtak Cho, Eung Nam Kim, Je Sung You, Minkyung Han, Yoo Seok Park

**Affiliations:** 1 Department of Emergency Medicine, Yonsei University College of Medicine, Seoul 03722, Republic of Korea; 2 Department of Emergency Medicine, Hanyang University Hospital, Seoul 04763, Republic of Korea; 3 Biostatistics Collaboration Unit, Department of Biomedical Systems Informatics, Yonsei University College of Medicine, Seoul 03722, Republic of Korea

**Keywords:** muscle mass index, neurological outcome, out-of-hospital cardiac arrest, sarcopenia, computed tomography

## Abstract

Muscle mass depletion is associated with unfavorable outcomes in many diseases. However, its relationship with cardiac arrest outcomes has not been explored. This retrospective single-center study determined the relationship between muscle mass depletion and the neurological outcomes of patients with out-of-hospital cardiac arrest (OHCA) by measuring muscle mass at various locations. Adult patients with OHCA, who were treated with target temperature management, and who underwent abdominal or chest computed tomography (CT) within 3 months of the cardiac arrest were included. Skeletal muscle index (SMI) was measured at the third lumbar vertebra (L3) level, psoas muscle, fourth thoracic vertebra (T4) level, and pectoralis muscle. The Youden index was used to determine a low SMI based on sex-specific cutoff values. The outcome variables were “good neurological outcome” and “survival” at hospital discharge. Multivariable analyses revealed that patients with low T4 SMI level were significantly associated with good neurological outcomes at hospital discharge (odds ratio = 0.26, 95% confidence interval: 0.07–0.88, *p* = 0.036). However, no significant differences were observed between good neurological outcomes and low SMI at the L3 level and psoas and pectoralis muscles; SMIs were not associated with survival at hospital discharge. T4 level SMI depletion was inversely associated with good neurological outcomes in patients with OHCA. Thoracic muscle depletion may be crucial for predicting the neurological outcomes in patients with OHCA and further investigation in larger prospective study is warranted.

## 1. Introduction

Out-of-hospital cardiac arrest (OHCA) is a critical situation presented in emergency departments. OHCA necessitates a substantial utilization of medical resources both during resuscitation and after the return of spontaneous circulation (ROSC). Due to the high prevalence of poor neurological outcomes, early prediction can facilitate decisions regarding the extent of treatment, such as targeted temperature management (TTM) [1,2]. Among various prognostic predictors of OHCA, factors related to metabolic conditions, including body mass index (BMI) and cholesterol, have gained attention [3,4]. However, BMI has several limitations; for example, it does not distinguish between fat and muscle, and disorders such as sarcopenic obesity may be underestimated or missed [5]. Therefore, an index measuring muscle quantity may be more accurate than BMI. Muscle mass measurement is an indirect method for predicting sarcopenia and can distinguish patients with similar BMIs based on muscle quantities [5,6].

Muscle mass measurement estimates muscle quantity and significantly correlates with muscle strength [7]. Assessment of skeletal muscle area (SMA) based on the cross-sectional area of a specific body location using magnetic resonance imaging and computed tomography (CT) is considered the gold standard [5]. Only one study has analyzed the association between muscle mass depletion and neurological outcomes of cardiac arrest [8]; however, the study was limited to patients with in-hospital cardiac arrest (IHCA), and muscle mass was assessed only at the third lumbar vertebra (L3) level using an abdominal CT. Although the skeletal muscle index (SMI) at the L3 level is the most common method for predicting sarcopenia, abdominal CT scanning, an essential part of this method, is not frequently used in patients with cardiac arrest. Accordingly, SMI at the pectoralis muscle and fourth thoracic vertebra (T4) level have been used in predicting other diseases, such as chronic obstructive pulmonary disease [9,10,11,12]. However, such studies on cardiac arrests are lacking.

Therefore, this study aimed to investigate the relationship between muscle mass depletion and neurological outcomes in patients with OHCA by measuring SMI at various locations and to determine the SMI that most accurately describes this relationship.

## 2. Materials and Methods

### 2.1. Settings and Study Population

This retrospective single-center study was conducted at a tertiary care hospital in the Republic of Korea. Adult patients (>18 years old) with OHCA who had undergone TTM between May 2012 and December 2021 were enrolled. Patients with available chest or abdominal CT scans within 3 months of cardiac arrest were included in the study, based on a previous study involving patients with IHCA [8]. Patients for whom TTM was discontinued, such as those with do-not-resuscitate orders or those with uncontrolled bleeding, were excluded. Additionally, patients who were transferred from the emergency department to another hospital were excluded, owing to missing outcome data, those with a cerebral performance category (CPC) score of 3–5 before cardiac arrest, and those with conditions affecting image clarity, such as muscle hematoma, were also excluded.

### 2.2. Post-Cardiac Arrest Care and TTM

All adult patients with OHCA and ROSC who were eligible for TTM underwent the standardized protocol of our institution. Patients who were unconscious and not able to follow verbal commands with more than 20 consecutive minutes of spontaneous circulation were considered eligible for the protocol. The target temperature was 33 °C before 2014 and chosen between 33 °C to 36 °C based on physician’s discretion after 2014. Cooling was discontinued after 24 h, followed by rewarming at a rate of 0.25 °C/h to achieve normothermia (37.0 °C). Normothermia was maintained for 48 h after the end of rewarming. TTM was conducted using Artic Sun^®^ Energy Transfer Pads™ (Medivance Corp, Louisville, KY, USA). 

Ventilator settings were adjusted to maintain SaO_2_ from 94% to 96% and PaCO_2_ from 40 to 45 mmHg to avoid hyperoxemia, hypocarbia, and hypercarbia. Blood pressure was corrected if systolic blood pressure was less than 90 mmHg. All patients received sedative and analgesic treatments. Bedside physical therapy was prescribed at admission.

### 2.3. Data Collection

Data including the patient age, sex, smoking status, weight, height, and comorbidities were collected from the electronic medical records. The Charlson Comorbidity Index (CCI) was calculated, and BMI was calculated as weight (kg) divided by height squared (m^2^). Resuscitation variables, such as witnessed collapse, location of arrest, bystander response, shockable rhythm, etiology of arrest, time from cardiac arrest to ROSC, application of percutaneous coronary intervention (PCI), and extracorporeal membrane oxygenation, were extracted from the emergency medical records [13,14]. Regarding unwitnessed cardiac arrest, the arrest time was calculated from the time of emergency call.

The primary outcome was a good neurological outcome, defined by a CPC score of 1 or 2 at hospital discharge [15,16]. Survival at hospital discharge was investigated as a secondary outcome. Ventilator-free days were also assessed for potential association between SMI and weaning of mechanical ventilation, which may have affected the outcomes of the study.

### 2.4. Muscle Mass Measurement

**Figure 1:**
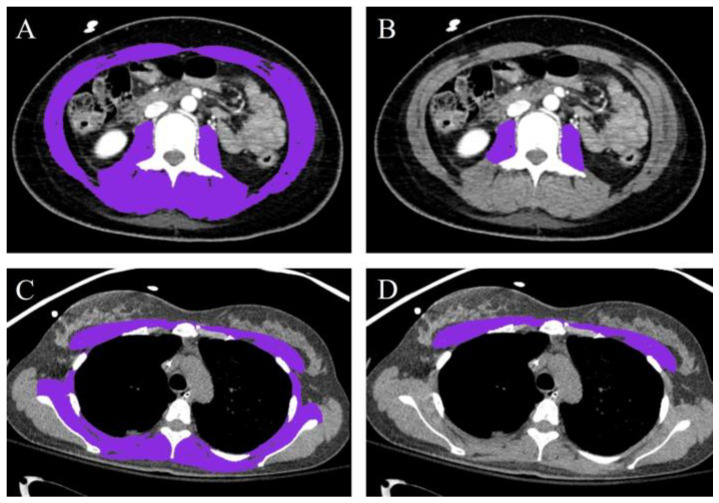
Cross-sectional CT images of the SMA indicated in purple. (**A**) L3 level SMA, (**B**) psoas muscle area, (**C**) T4 level SMA, and (**D**) pectoralis muscle area. Abbreviations: CT, computed tomography; L3, the third lumbar vertebra; SMA, skeletal muscle area; T4, the fourth thoracic vertebra.

CT scans were acquired with a multidetector CT scanner (SOMATOM Force, SIMENS Healthneers, Germany). CT technical parameters included syngo CT VB20A (Software Versions), 120 kV (tube voltage), 0.6 mm, 192 rows (detector configuration), tube current modulation, 0.25 s/rotation (gantry rotation), and a 3 mm reconstruction thickness. Chest CT was performed from the lung apex to the diaphragm, and abdominal CT was performed from the diaphragmatic dome to the symphysis pubis. The following four skeletal muscles were assessed: L3 level SMA, psoas muscle area, T4 level SMA, and pectoralis muscle area. SMA at the L3 level is the sum of muscles identified on CT scans at the L3 inferior endplate level, including the paraspinal, abdominal wall, and psoas muscles [5,10,17]. The psoas muscle area was measured at the same level as that of the L3 SMA [5,18,19]. The T4 level SMA included the pectoralis, intercostalis, paraspinal, serratus, and latissimus muscles measured at the middle of the fourth vertebral body [5,20,21,22]. The pectoralis muscle area was defined as the sum of the bilateral pectoralis major and minor muscles at the level just above the aortic arch [5,23,24]. Skeletal muscles were demarcated using Hounsfield units, ranging from −29 to 150 [25]. All images were semi-automatically analyzed using the Aview^®^ system (v 1.1.38.6, Coreline Soft Inc., Seoul, Republic of Korea) and reviewed by two emergency medicine doctors (Figure 1). SMI was calculated as SMA (cm^2^)/BMI (kg/m^2^) and normalized to the BMI [5,26,27,28].

### 2.5. Statistical Analyses

Categorical variables are presented as frequency (%), while continuous variables are presented as mean ± standard deviation or median with an interquartile range. The normality of the distribution was examined using the Shapiro–Wilk test. Baseline characteristics were compared between good and bad neurological outcome groups using the Mann–Whitney *U* test or Student’s *t*-test for continuous variables and the chi-squared test or Fisher’s exact test for categorical variables, as appropriate. To assess the relationship between the outcomes and SMI, each sex-specific cutoff value of SMI was set for good neurological outcome and survival. Although the recommended cutoff value for sarcopenia is usually set at −2 standard deviations in healthy young adults, a universal cutoff value for SMI normalized by BMI has not been established [5]. Therefore, to select the optimal cutoff values with the best discriminative performance for predicting outcomes, a receiver operating characteristic analysis was performed. Based on the Youden index, each sex-specific cutoff value for SMI was selected where the Youden index was defined as sensitivity + specificity of −1. A univariate logistic regression analysis was performed using demographic variables, resuscitation variables, and SMI to evaluate the risk factors associated with the primary or secondary outcomes. Next, a multivariable logistic regression analysis was performed using variables that had a *p* value < 0.1 in the univariate logistic regression. Multicollinearity was eliminated using a generalized variance inflation factor. The correlation between ventilator-free days and SMI was analyzed using Kendall’s tau. All reported *p* values were two-sided, and statistical significance was set at *p* < 0.05. Statistical analyses were performed using R software (version 4.3.0, http://www.R-project.org, accessed on 3 March 2023).

## 3. Results

**Figure 2:**
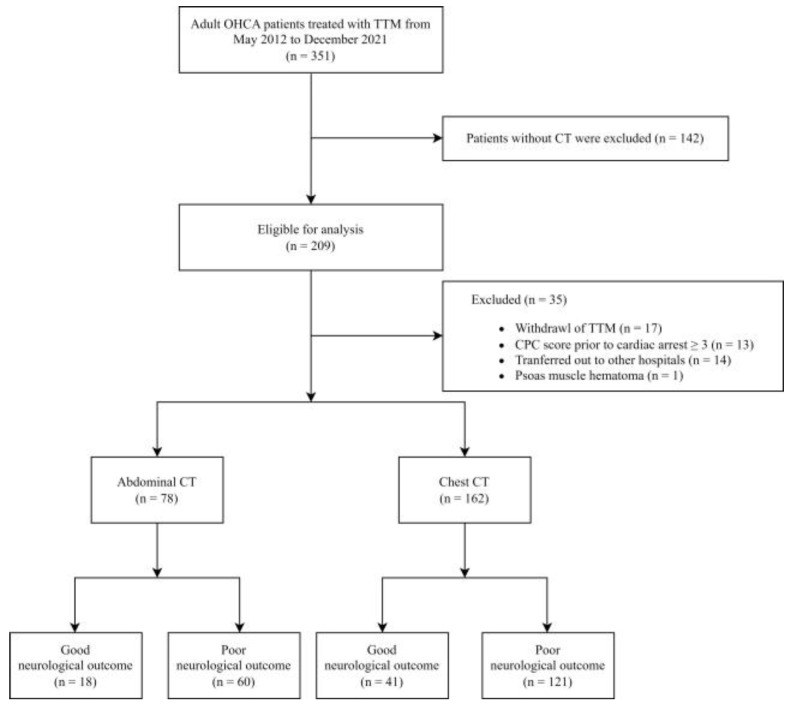
Patient flow diagram. Abbreviations: CPC, cerebral performance category; CT, computed tomography; OHCA, out-of-hospital cardiac arrest; TTM, targeted temperature management.

Between May 2012 and December 2021, 351 patients with OHCA were treated with TTM in the emergency department. After excluding patients without CT scans, 209 patients were eligible for analysis, among whom abdominal and chest CT scans were performed in 78 and 162 patients, respectively, within 3 months of cardiac arrest. Of the 78 patients who underwent abdominal CT, 18 exhibited good neurological outcomes at discharge, whereas among the 162 patients who underwent chest CT, 41 exhibited good neurological outcomes (Figure 2).

Baseline characteristics of patients who underwent abdominal and chest CT compared according to their neurological outcomes and survival at hospital discharge are presented in Table 1. Among the patients who underwent abdominal CT, those with good neurological outcomes had differences in the location of cardiac arrest (*p* = 0.007), shockable rhythm (*p* < 0.001), cardiogenic arrest (*p* < 0.001), and PCI (*p* = 0.012). Patients who survived until discharge showed statistically significant differences only in witnessed arrest (*p* = 0.013) and shockable rhythm (*p* = 0.038). Baseline characteristics of total patients with OHCA are presented in Appendix A.

Among the patients who underwent chest CT, the good neurological outcome group was significantly associated with male sex (*p* = 0.007) and a lower CCI score (*p* = 0.013). Additionally, they had differences in resuscitation variables compared with the bad neurological outcomes group in witnessed arrest (*p* = 0.031), location of arrest (*p* = 0.002), bystander cardiopulmonary resuscitation (CPR) (*p* = 0.014), shockable rhythm (*p* < 0.001), cardiogenic arrest (*p* < 0.001), arrest time (*p* = 0.001), and PCI (*p* < 0.001). The patients who survived until discharge were younger (*p* = 0.040) and had lower CCI scores (*p* = 0.004). The survival group had a higher incidence of witnessed cardiac arrest (*p* = 0.001), bystander CPR (*p* = 0.013), shockable rhythm (*p* < 0.001), and cardiogenic arrest (*p* = 0.005). The arrest time was significantly shorter in the survival group (*p* = 0.005).

Kendall’s tau correlation analysis on the relationship between ventilator-free days and SMIs showed weak or no correlations for L3/BMI (τ = 0.267, *p* = 0.002), psoas/BMI (τ = 0.260, *p* = 0.003), T4/BMI (τ = 0.113, *p* = 0.059), and pectoralis/BMI (τ = 0.133, 0.026).

The sex-specific cutoff values for good neurological outcomes and survival are presented in Table 2. The patients were divided into low- or high-SMI groups based on these values. Among the four evaluated SMIs, T4/BMI and pectoralis/BMI were associated with neurological outcomes (*p* < 0.001 and *p* = 0.001, respectively). However, the L3/BMI was not associated with survival until discharge (Table 3).

In the multivariable analyses, age and sex were included because of their clinical significance. Age, sex, CCI, witnessed arrest, location of arrest, bystander CPR, shockable rhythm, cardiogenic cause, arrest time, and PCI were included as confounding variables in the multivariable logistic regression analysis for good neurological outcomes in the patients who underwent a chest CT. Bystander CPR (*p* = 0.018), arrest time (*p* = 0.023), and low T4/BMI (*p* = 0.021) were significantly associated with good neurological outcomes in the multivariable analysis. The association between low pectoralis/BMI and good neurological outcome (*p* = 0.222) was lost after adjusting for the confounding variables. The confounding variables for survival at discharge in the chest CT group were similar to those of the primary outcome, except for the location of arrest and PCI. Both thoracic muscle indices had no predictive value for survival at hospital discharge in the multivariable analysis. A low psoas/BMI, which had statistical significance in the univariable analysis for survival to discharge, failed to show any association after adjusting for the confounding variables (Table 4).

## 4. Discussion

To the best of our knowledge, this study was the first to investigate the relationship between various muscle measurements at various locations and neurological outcomes in patients with OHCA. The results showed that only T4/BMI was independently associated with neurological outcomes in patients with OHCA. Pectoralis/BMI was statistically significant in the univariable analysis, although not after adjusting for other covariates.

This result differs from that of a previous study involving patients with IHCA, which showed an association between SMI at the L3 level and long-term neurological outcomes [8]. These differences may be due to differences in the OHCA and IHCA characteristics. Patients with IHCA may have more comorbidities and a poor nutritional status, which can be observed in the prevalence of patients with cancer, 53.2% in the study by Hong et al. [8] and 4.5% in this study. Overall, 27% of patients with OHCA who were treated with TTM had good neurological outcomes at hospital discharge. These results were not significantly different from those of a previous study (approximately 30.5%) for patients with OHCA in the Republic of Korea [28]. Regarding cardiac arrest characteristics, bystander CPR and arrest time were associated with good neurological outcomes. The association of BMI with cardiac arrest outcomes remains debatable and was not observed in this study [3].

The European Working Group on Sarcopenia in Older People proposed a revised definition of sarcopenia in 2018, using low muscle strength as the primary parameter. Probable sarcopenia is identified by low muscle strength and diagnosed when muscle quantity or quality is low [5]. However, muscle strength measurements are difficult in clinical settings, particularly in the emergency department. In previous studies, skeletal muscle mass measurements have been used to indirectly diagnose sarcopenia, owing to its positive correlation with muscle strength [29,30]. Considering that muscle mass is related to body size, BMI was used in this study to mitigate the association between SMA and body size. Due to its positive correlation with body size, previous studies have proposed muscle mass indices to be adjusted for height squared, weight, or BMI [5]. Since the proposal of the SMA/BMI index by the Foundation for the National Institutes of Health Sarcopenia Project, it has gained much interest [26]. Although studies have used height squared to normalize muscle mass, several studies have suggested that muscle mass normalized by height squared can underestimate sarcopenia prevalence in individuals with obesity and that BMI is a better metric [27,28]. Therefore, BMI was used in this study.

Our findings suggest that the thoracic muscles may have more important functional and predictive values than those of other cross-sectional areas in patients with OHCA; however, the underlying mechanism remains unclear. Previous studies have shown that patients with greater thoracic muscle area have better lung function and physical activity in respiratory diseases [23,31]. Furthermore, the importance of respiratory muscles in the outcomes of COVID-19 and idiopathic pulmonary fibrosis has been investigated [21,32]. This suggests the need for a disease-specific approach in muscle mass assessment. For example, fat-free mass in the limbs does not correlate with that of the entire body [33]. Additionally, a recent study reported a significant association between SMI at the L3 level and survival in patients with multiple traumas but not with SMI at the T4 level [34]. Although previous studies have suggested strong correlations between total muscle mass and the L3, T4, and pectoralis muscles, caution should be exercised when determining the level of cross-sectional area to be used [20,33,35]. Further studies are needed to investigate the role of thoracic muscles in patients with cardiac arrest.

Our study revealed conflicting results between the T4/BMI and pectoralis/BMI, although both consist of the thoracic muscles. This may be due to the fact that the pectoralis muscles are not fully representative of the respiratory muscles; they only consist of pectoralis major and minor muscles, while the T4/SMI includes other important breathing muscles such as the intercostalis muscles. Given its primary role in upper arm exercises, the pectoralis major muscle, which contributes to the majority of the pectoralis muscle areas, may have not reflected the outcomes of patients with OHCA as well as the T4 level SMI [20,36]. However, the relationship between ventilator weaning and respiratory muscles of patients with OHCA should be further investigated, considering that our results did not show any strong relationship between SMIs and ventilator-free days. 

The time window of 3 months was determined based on a similar study involving patients with IHCA [8]. However, due to its relatively long duration, some patients may have accurately reflected pre-cardiac arrest muscle mass, while for others, the CT scans could have been significantly influenced by post-arrest conditions. Nonetheless, considering that the majority of patients underwent the examination shortly after arrest, it can still be assumed that most scans predominantly represent pre-cardiac arrest muscle mass. Nevertheless, there may be a need for prospective studies conducted at more standardized times to measure muscle mass.

### Limitations

This study has several limitations. First, this was a single-center retrospective study with possible bias. However, a consistent protocol was followed throughout the treatment, owing to the single-center design. Second, the outcomes were analyzed at hospital discharge without long-term data, although studies have reported that outcomes at discharge do not differ from long-term outcomes [37,38]. Third, CT images were not reviewed by a specialized radiologist. Two experienced emergency medicine doctors reviewed all images to minimize potential errors. Fourth, the post-cardiac arrest management protocol may differ in aspects such as target temperature within the study population, which could have influenced the results. However, due to the retrospective nature of the study, it was difficult to control this parameter, and the sample size was too small to conduct subgroup analysis. Moreover, post-cardiac arrest conditions may have influenced muscle mass due to the relatively long-time window between arrest and CT scan. Finally, we used cutoff values based on our study population. The cutoff values for sarcopenia should be calculated based on a healthy population; however, no optimal cutoff values for these indices have been established. Therefore, the cutoff values presented in this study should be implemented cautiously in the general population.

## 5. Conclusions

SMI at the T4 level was associated with neurological outcomes in patients with OHCA. However, SMIs at the L3 level and psoas and pectoralis muscles showed no associations with OHCA outcomes. Thus, low thoracic muscle may be associated with neurological outcomes in patients with OHCA and should be further investigated in a large prospective study.

## Figures and Tables

**Table 1:** Baseline characteristics of patients with OHCA.

Abdominal CT Group (n = 78)						
	Good Neurological Outcome (n = 18)	Poor Neurological Outcome (n = 60)	*p* Value	Survival (n = 41)	Death (n = 37)	*p* Value
Age	58.5 [48.0–66.0]	65.0 [52.0–74.0]	0.267	62.0 [47.0–69.0]	65.0 [60.0–75.0]	0.064
Men	15 (83.3)	39 (65.0)	0.235	32 (78.0)	22 (59.5)	0.126
BMI (kg/m^2^)	23.0 ± 3.9	24.1 ± 4.1	0.310	23.4 ± 4.0	24.3 ± 4.2	0.336
Smoking			0.357			0.710
Current	6 (33.3)	12 (20.0)		11 (26.8)	7 (18.9)	
Ex-smoker	3 (16.7)	7 (11.7)		5 (12.2)	5 (13.5)	
Non-smoker	9 (50.0)	41 (68.3)		25 (61.0)	25 (67.6)	
CCI	2.5 [1.0–4.0]	3.5 [2.0–6.5]	0.146	3.0 [1.0–5.0]	4.0 [2.0–8.0]	0.146
Witnessed arrest	14 (77.8)	38 (63.3)	0.392	33 (80.5)	19 (51.4)	0.013
Location			0.007			0.376
Residence	3 (16.7)	34 (56.7)		17 (41.5)	20 (54.1)	
Other	15 (83.3)	26 (43.3)		24 (58.5)	17 (45.9)	
Bystander CPR	14 (77.8)	35 (58.3)	0.223	26 (63.4)	23 (62.2)	1.000
Shockable rhythm	11 (61.1)	9 (15.0)	<0.001	15 (36.6)	5 (13.5)	0.038
Cardiogenic cause	14 (77.8)	17 (28.3)	<0.001	19 (46.3)	12 (32.4)	0.307
Arrest time ^a^ (mins)	22.5 [13.0–41.0]	35.5 [22.5–44.0]	0.100	32.0 [21.0–42.0]	33.0 [18.0–44.0]	0.984
PCI	7 (38.9)	6 (10.0)	0.012	9 (22.0)	4 (10.8)	0.311
ECMO			0.114			0.272
ECPR	0 (0.0)	1 (1.7)		1 (2.4)	0 (0.0)	
after ROSC	3 (16.7)	2 (3.3)		4 (9.8)	1 (2.7)	
No ECMO	15 (83.3)	57 (95.0)		36 (87.8)	36 (97.3)	
Target body temperature			0.545			0.955
33 °C	4 (22.2)	20 (33.3)		12 (29.3)	12 (32.4)	
36 °C	14 (77.8)	40 (66.7)		29 (70.7)	25 (67.6)	
NSE	37.7 [17.0–50.9]	198.5 [55.4–300.0]	0.001	61.0 [37.7–300.0]	198.5 [47.6–300.0]	0.350
Initial PaO_2_ (mmHg)	137.7 [62.8–235.6]	102.5 [72.5–162.1]	0.294	106.4 [75.7–187.4]	105.6 [64.8–177.5]	0.503
PaO_2_ after 4 h (mmHg)	159.2 [73.6–179.5]	121.7 [89.1–241.8]	0.631	164.2 [94.0–238.5]	116.0 [83.0–217.0]	0.376
Initial PaCO_2_ (mmHg)	46.0 ± 19.5	72.4 ± 28.7	<0.001	57.3 [41.1–80.6]	67.0 [52.5–84.8]	0.596
PaCO_2_ after 4 h (mmHg)	37.4 [33.7–43.2]	47.9 [36.8–56.6]	0.029	41.0 [33.7–51.9]	45.4 [39.3–55.6]	0.211
Time from arrest to CT (days)	0.0 [0.0–0.0]	0.0 [0.0–0.0]	0.077	0.0 [0.0–0.0]	0.0 [0.0–0.0]	0.625
**Chest CT Group (n = 162)**						
	**Good Neurological Outcome** **(n = 41)**	**Poor Neurological Outcome** **(n = 121)**	***p*** **Value**	**Survival** **(n = 91)**	**Death** **(n = 71)**	***p*** **Value**
Age	59.0 [47.0–66.0]	64.0 [49.0–74.0]	0.085	59.0 [43.5–70.5]	65.0 [55.0–74.0]	0.040
Men	36 (87.8)	77 (63.6)	0.007	68 (74.7)	45 (63.4)	0.165
BMI (kg/m^2^)	23.3 [22.0–26.0]	23.5 [21.1–26.2]	0.767	23.9 ± 4.1	23.3 ± 4.6	0.357
Smoking			0.317			0.174
Current	14 (34.1)	29 (24.0)		29 (31.9)	14 (19.7)	
Ex-smoker	7 (17.1)	17 (14.0)		14 (15.4)	10 (14.1)	
Non-smoker	20 (48.8)	75 (62.0)		48 (52.7)	47 (66.2)	
CCI	2.0 [1.0–4.0]	4.0 [1.0–6.0]	0.013	3.0 [0.5–5.0]	4.0 [2.0–7.5]	0.004
Witnessed arrest	33 (80.5)	73 (60.3)	0.031	70 (76.9)	36 (50.7)	0.001
Location			0.002			0.115
Residence	9 (22.0)	63 (52.1)		35 (38.5)	37 (52.1)	
Other	32 (78.0)	58 (47.9)		56 (61.5)	34 (47.9)	
Bystander CPR	34 (82.9)	73 (60.3)	0.014	68 (74.7)	39 (54.9)	0.013
Shockable rhythm	29 (70.7)	19 (15.7)	<0.001	40 (44.0)	8 (11.3)	<0.001
Cardiogenic cause	33 (80.5)	43 (35.5)	<0.001	52 (57.1)	24 (33.8)	0.005
Arrest time ^a^ (mins)	20.0 [13.0–28.0]	33.0 [20.0–44.0]	0.001	25.0 [15.0–39.0]	37.0 [21.0–45.0]	0.005
PCI	15 (36.6)	9 (7.4)	<0.001	18 (19.8)	6 (8.5)	0.073
ECMO			0.278			0.454
ECPR	1 (2.4)	1 (0.8)		2 (2.2)	0 (0.0)	
after ROSC	4 (9.8)	5 (4.1)		5 (5.5)	4 (5.6)	
No ECMO	36 (87.8)	115 (95.0)		84 (92.3)	67 (94.4)	
Target body temperature			0.035			0.960
33 °C	7 (17.1)	44 (36.4)		28 (30.8)	23 (32.4)	
36 °C	34 (82.9)	77 (63.6)		63 (69.2)	48 (67.6)	
NSE	24.6 [17.6–37.7]	206.5 [48.5–300.0]	<0.001	44.8 [27.1–221.1]	235.6 [55.4–300.0]	0.003
Initial PaO_2_ (mmHg)	106.4 [68.3–212.2]	99.4 [64.8–160.8]	0.289	92.4 [65.8–184.9]	115.1 [67.5–178.4]	0.510
PaO_2_ after 4 h (mmHg)	134.9 [87.5–193.3]	135.8 [88.2–230.4]	0.739	140.2 [88.0–204.3]	123.9 [85.6–229.0]	0.939
Initial PaCO_2_ (mmHg)	44.6 [30.3–59.4]	71.4 [52.9–86.4]	<0.001	58.0 [41.4–78.8]	68.8 [52.0–87.0]	0.038
PaCO_2_ after 4 h (mmHg)	41.0 [34.3–48.7]	46.0 [36.7–55.1]	0.051	45.8 [36.3–52.5]	45.1 [35.6–54.9]	0.991
Time from arrest to CT (days)	0.0 [0.0–0.0]	0.0 [0.0–0.0]	0.077	0.0 [0.0–0.0]	0.0 [0.0–0.0]	0.625

^a^ For unwitnessed cardiac arrest, arrest time was calculated from the time of the emergency call. Data are presented as the mean ± standard deviation, median [interquartile range], or number (percentage). Percentages may not total up to100 due to rounding. Abbreviations: BMI, body mass index; CCI, Charlson Comorbidity Index; CPR, cardiopulmonary resuscitation; CT, computed tomography; ECMO, extracorporeal membrane oxygenation; ECPR, extracorporeal cardiopulmonary resuscitation; OHCA, out-of-hospital cardiac arrest; PCI, percutaneous coronary intervention; ROSC, return of spontaneous circulation.

**Table 2:** Cutoff values for the SMI of L3/BMI, psoas/BMI, T4/BMI, and pectoralis/BMI.

	Good Neurological Outcome		Survival	
	Cutoff Value		Cutoff Value	
	Men	Women	Men	Women
L3/BMI	5.8994	3.5512	6.489	4.6331
Psoas/BMI	0.7703	0.3152	0.7703	0.5832
T4/BMI	5.4658	4.9739	5.7717	4.9545
Pectoralis/BMI	2.1648	1.531	2.2767	1.7563

Abbreviations: L3/BMI, the third lumbar vertebra level muscle area/body mass index; pectoralis/BMI, pectoralis muscle area/body mass index; psoas/BMI, psoas muscle area/body mass index; SMI, skeletal muscle index; T4/BMI, the fourth thoracic vertebra level muscle area/body mass index.

**Table 3:** Low SMI outcomes.

	Good Neurological Outcome (n = 18)	Poor Neurological Outcome (n = 60)	*p* Value	Survival (n = 41)	Death (n = 37)	*p* Value
Low L3/BMI	6 (33.3)	28 (46.7)	0.466	25 (61.0)	29 (78.4)	0.156
Low psoas/BMI	6 (33.3)	29 (48.3)	0.394	22 (53.7)	30 (81.1)	0.020
	**Good Neurological Outcome (n = 41)**	**Poor Neurological Outcome (n = 121)**	***p* Value**	**Survival** **(n = 91)**	**Death** **(n = 71)**	***p* Value**
Low T4/BMI	6 (14.6)	68 (56.2)	<0.001	35 (38.5)	48 (67.6)	<0.001
Low pectoralis/BMI	14 (34.1)	78 (64.5)	0.001	49 (53.8)	53 (74.6)	0.011

Values are expressed as numbers (percentages). Percentages may not total up to 100 due to rounding. Abbreviations: L3/BMI, the third lumbar vertebra level muscle area/body mass index; pectoralis/BMI, pectoralis muscle area/body mass index; psoas/BMI, psoas muscle area/body mass index; SMI, skeletal muscle index; T4/BMI, the fourth thoracic vertebra level muscle area/body mass index.

**Table 4:** Multivariable logistic analysis for good neurological outcomes and survival at hospital discharge.

	Good Neurological Outcome				Survival					
	Adjusted OR (95% CI)	*p* Value	Adjusted OR (95% CI)	*p* Value	Adjusted OR (95% CI)	*p* Value	Adjusted OR (95% CI)	*p* Value	Adjusted OR (95% CI)	*p* Value
Low psoas/BMI					0.38 (0.11–1.19)	0.102				
Low T4/BMI	0.22 (0.05–0.76)	0.021					0.56 (0.24–1.31)	0.182		
Low pectoralis/ BMI			0.49 (0.15–1.55)	0.222					0.80 (0.33–1.96)	0.620
Age	1.00 (0.95–1.04)	0.937	1.00 (0.96–1.05)	0.926	0.98 (0.95–1.01)	0.300	1.00 (0.97–1.03)	0.795	1.00 (0.97–1.03)	0.886
Men	1.15 (0.28–5.09)	0.845	1.94 (0.55–7.82)	0.318	1.79 (0.58–5.71)	0.315	0.99 (0.41–2.36)	0.981	1.13 (0.49–2.64)	0.769
CCI	0.97 (0.72–1.28)	0.818	0.91 (0.68–1.19)	0.498			0.84 (0.68–1.02)	0.089	0.81 (0.66–0.99)	0.039
Witnessed arrest	1.85 (0.51–7.37)	0.359	1.93 (0.57–7.14)	0.302	3.42 (1.18–10.68)	0.027	3.13 (1.38–7.38)	0.007	3.40 (1.52–7.94)	0.004
Location (Residence)	0.47 (0.14–1.44)	0.193	0.46 (0.14–1.40)	0.182						
Bystander CPR	5.15 (1.46–22.66)	0.018	4.76 (1.40–19.97)	0.020			2.09 (0.94–4.71)	0.071	2.02 (0.92–4.53)	0.082
Shockable rhythm	3.31 (0.93–12.70)	0.068	3.20 (0.91–12.09)	0.074	2.34 (0.70–8.71)	0.179	3.72 (1.15–12.95)	0.032	3.77 (1.18–12.94)	0.029
Cardiogenic cause	2.72 (0.65–11.57)	0.166	2.82 (0.69–11.51)	0.142			1.29 (0.50–3.34)	0.597	1.28 (0.50–3.28)	0.603
Arrest time	0.96 (0.93–0.99)	0.023	0.96 (0.93–0.99)	0.020			0.97 (0.95–0.99)	0.011	0.97 (0.95–0.99)	0.011
PCI	4.20 (1.04–19.75)	0.053	3.84 (0.96–17.76)	0.067						

This table presents the results of multivariable logistic analysis of SMIs with clinical significance to the outcomes in the univariable analysis. Column 1 represents the results of low T4/BMI and covariates, and column 2 represents the results of low pectoralis/BMI and covariates to good neurological outcome. Columns 3–5 represent the results of low psoas/BMI, low T4/BMI, and low pectoralis/BMI with covariates to survival, respectively. Abbreviations: BMI, body mass index; CCI, Charlson Comorbidity Index; CI, confidence interval; CPR, cardiopulmonary resuscitation; OR, odds ratio; PCI, percutaneous coronary intervention; pectoralis/BMI, pectoralis muscle area/body mass index; psoas/BMI, psoas muscle area/body mass index; SMI, skeletal muscle index; T4/BMI, the fourth thoracic vertebra level muscle area/body mass index.

## Data Availability

The datasets used during the current study are available from the corresponding author upon reasonable request.

## Appendix A

**Table A1:** Baseline characteristics of total patients with OHCA.

	Total (n = 174)	Good Neurological Outcome (n = 101)	Poor Neurological Outcome (n = 73)	*p* Value
Age	63.0 [48.0–72.0]	59.0 [45.0–70.0]	65.0 [55.0–74.0]	0.019
Men	123 (70.7)	77 (76.2)	46 (63.0)	0.085
BMI (kg/m^2^)	23.4 [21.3–25.9]	23.7 [21.4–26.3]	22.9 [21.0–25.3]	0.517
Smoking				0.222
Current	47 (27.0)	32 (31.7)	15 (20.5)	
Ex-smoker	27 (15.5)	16 (15.8)	11 (15.1)	
Non-smoker	100 (57.5)	53 (52.5)	47 (64.4)	
CCI	3.0 [1.0–5.0]	3.0 [0.0–5.0]	4.0 [2.0–7.0]	0.004
Witnessed arrest	115 (66.1)	38 (52.1)	77 (76.2)	0.002
Location				0.153
Residence	76 (43.7)	39 (38.6)	37 (50.7)	
Other	98 (56.3)	62 (61.4)	36 (49.3)	
Bystander CPR	115 (66.1)	74 (73.3)	41 (56.2)	0.029
Shockable rhythm	54 (31.0)	45 (44.6)	9 (12.3)	<0.001
Cardiogenic cause	84 (48.3)	59 (58.4)	25 (34.2)	0.003
Arrest time ^a^ (mins)	28.5 [17.0–43.0]	26.0 [15.0–40.0]	37.0 [20.0–45.0]	0.014
PCI	28 (16.1)	21 (20.8)	7 (9.6)	0.076
ECMO				0.475
ECPR	2 (1.1)	2 (2.0)	0 (0.0)	
after ROSC	10 (5.7)	6 (5.9)	4 (5.5)	
No ECMO	162 (93.1)	93 (92.1)	69 (94.5)	

^a^ For unwitnessed cardiac arrest, arrest time was calculated from the time of the emergency call. Data are presented as the mean ± standard deviation, median [interquartile range], or number (percentage). Percentages may not total 100 due to rounding. Abbreviations: BMI, body mass index; CCI, Charlson Comorbidity Index; CPR, cardiopulmonary resuscitation; ECMO, extracorporeal membrane oxygenation; ECPR, extracorporeal cardiopulmonary resuscitation; OHCA, out-of-hospital cardiac arrest; PCI, percutaneous coronary intervention; ROSC, return of spontaneous circulation.
